# Genetic Algorithm for the Design of Electro-Mechanical Sigma Delta Modulator MEMS Sensors

**DOI:** 10.3390/s111009217

**Published:** 2011-09-27

**Authors:** Reuben Wilcock, Michael Kraft

**Affiliations:** School of Electronics and Computer Science, University of Southampton, Highfield, Southampton SO17 1BJ, UK; E-Mail: rw3@ecs.soton.ac.uk

**Keywords:** genetic algorithm (GA), sigma delta modulator (ΣΔM), micro-electro-mechanical systems (MEMS), gyroscope, accelerometer

## Abstract

This paper describes a novel design methodology using non-linear models for complex closed loop electro-mechanical sigma-delta modulators (EMΣΔM) that is based on genetic algorithms and statistical variation analysis. The proposed methodology is capable of quickly and efficiently designing high performance, high order, closed loop, near-optimal systems that are robust to sensor fabrication tolerances and electronic component variation. The use of full non-linear system models allows significant higher order non-ideal effects to be taken into account, improving accuracy and confidence in the results. To demonstrate the effectiveness of the approach, two design examples are presented including a 5th order low-pass EMΣΔM for a MEMS accelerometer, and a 6th order band-pass EMΣΔM for the sense mode of a MEMS gyroscope. Each example was designed using the system in less than one day, with very little manual intervention. The strength of the approach is verified by SNR performances of 109.2 dB and 92.4 dB for the low-pass and band-pass system respectively, coupled with excellent immunities to fabrication tolerances and parameter mismatch.

## 1. Introduction

Embedding a micromachined sensing element in a closed loop, force feedback system is a technique commonly used to realise high performance MEMS (micro-electro-mechanical systems) sensors due to the many advantages attainable in terms of better linearity, increased dynamic range and bandwidth, and reduced parameter sensitivity to fabrication tolerances. In particular, MEMS inertial sensors employing a capacitive sensing element incorporated in sigma-delta modulator (ΣΔM) control systems with electrostatic feedback have gained popularity in the past due to their direct digital output signal, and avoidance of potential electro-static instability (due to the ‘pull-in’ effect). Earlier work used the micro-machined sensing element as the sole loop filter, and, since the sensing element is typically a second order mass-damper-spring low-pass filter, this resulted in a second order electro-mechanical ΣΔM (EMΣΔM) [1–3]. It is well known from purely electronic ΣΔM, used for analogue-to-digital conversion that such a second order system suffers from relatively high quantisation noise, idle tones and deadzones [4]; additionally, the micromachined sensing element represents two leaky integrators with low steady-state gain further reducing the noise shaping ability. It is therefore difficult to attenuate the quantization noise level below other noise sources originating from the electronic pick-off circuitry and the sensing element itself (Brownian noise). To address these shortcomings, recently, several research groups have designed and implemented EMΣΔM in which the sensing element is cascaded with an electronic filter comprising several integrators (or resonators, for band-pass EMΣΔMs); this has been successfully applied to MEMS accelerometers [5–8], and to control the sense mode of MEMS gyroscopes [9–12] resulting in far superior noise shaping abilities. The architectures are inspired by high order electronic analogue-to-digital ΣΔMs but these cannot be simply transferred to EMΣΔM due to the nature of the micromachined sensing element, which has an inaccessible internal node. In the past we have investigated several such architectures for MEMS accelerometers and gyroscopes [13,14].

Linearized analytical models for ΣΔMs are described in for example [15] and employed extensively in the design of analogue-to-digital ΣΔMs [16] to accurately predict performance. However, they have limited use in predicting the performance and stability of realistic EMΣΔMs systems for two reasons. Firstly, due to already having a second order sensor in the loop, high performance EMΣΔMs require a high overall loop order for which stability becomes a greater concern and the linearisation of the quantizer a less reasonable assumption [17]. Secondly, in contrast to purely electronic ΣΔMs, a strong non-linear term is introduced in EMΣΔMs due to the dependence of the feedback force on the sensor mass position and this has serious implications on stability and performance, which cannot be predicted with a linear model [8]. The solution is to use non-linear analysis for the design of EMΣΔMs but to date there has been no satisfactory analytical approach.

In this work we present a novel design methodology for EMΣΔM based on genetic algorithms (GA) and Monte Carlo simulations, both using accurate non-linear models. Genetic algorithms are based on the mechanics of natural selection and genetics combining the fittest individuals in the population in order to search for the best solution [18]. These evolutionary based techniques are excellent for particularly complex problems where they are capable of finding good solutions in a short period of time [19]. A typical GA consists of several stages including chromosome representation, initial population generation, evaluation of a fitness function followed by crossover and mutation. Once an initial population consisting of a number of individuals (parameter sets) has been randomly chosen, based on the fitness function the fittest individuals are selected and combined to produce a new offspring. As in real organisms, combination of two individuals will often produce offspring that are better adapted to the environment, thus having a better fitness score. A small mutation probability is then added to the new offspring, again copying nature and ensuring a diverse search of the gene space. The process is repeated over the whole population for a large number of generations and the result is a final population with a high fitness score. An elite preservation strategy can also be employed which ensures that a certain number of elite individuals are carried forth in each generation. In this way, good solutions found early on in the process will never be lost unless replaced by a better solution.

In this work the genetic algorithm used is the gamultiobj() function in Matlab which is variant on the Non-dominated Sorting Genetic Algorithm-II (NGSA-II) [20]. Numerous parameters governing the operation of this function can be tailored to its particular use. In the examples in this paper, the ‘EliteCount’ parameter was set to ‘2’ meaning that the best two individuals would always be carried forward. Mutation options specify the small random changes that the GA makes in the individuals to allow a broader search space, and in this work the ‘MutationFcn’ was set to ‘mutationuniform’. Uniform mutation involves first selecting a fraction of the design parameters for mutation and then replacing each designated parameter by a random number selected uniformly from the range for that entry. Crossover options specify how the GA combines two individuals, or parents, to form a next generation child individual. In this work the ‘CrossoverFunction’ was set to ‘crossoverscattered’ resulting in the parameter crossover being defined by a random binary vector. In this case, if the vector bit is set to ‘1’ the child gene (*i.e.*, parameter) comes from the first parent and vice versa. An initial population function was written and used to randomly generate the first population with a uniform distribution within the specified constraints.

As part of the overall design methodology, the GA facilitates multi-objective optimisation for the design of low-pass or band-pass EMΣΔM with a wide range of orders and with any architecture. Since the result of the optimisation is a large number of equally optimal solutions the design procedure subsequently carries out a robustness analysis based on statistical simulations to ensure stability of the design in the presence of fabrication tolerances, which can be substantial for micromachined sensing elements. Although numerous methods exist for output variation estimation, e.g., [21], in this work a Monte Carlo approach has been used due to its popularity and ease of implementation. The robustness analysis is a key contribution of this work, and helps ensure manufacturability and hence improve the yield of realised designs.

This paper is organised as follows: Section 2 describes the developed GA process in general; Section 3 gives an example for the design of a 5th order EMΣΔM MEMS accelerometer; Section 4 gives a second example of a band-pass EMΣΔM for a MEMS gyroscope; in Section 5 the design approach is discussed and in Section 6 conclusions are drawn.

## 2. Genetic Algorithm for High Order Electro-Mechanical Sigma Delta Modulators

**Figure 1.**
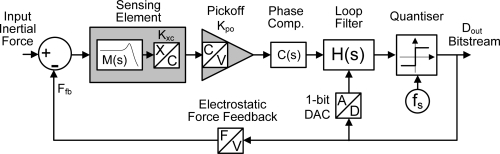
Block diagram of an electro-mechanical sigma-delta modulator.

An EMΣΔM consists of the following building blocks: (i) the micromachined sensing element; (ii) the pick-off circuit that capacitively measures the displacement of the proof mass in response to an inertial force and converts it to a voltage; (iii) a phase compensator (which may not be required if the sensing element is overdamped); (iv) an electronic loop filter comprising several integrators and minor feedback or feedforward loops; (v) a clocked one bit quantizer; (vi) a feedback block that converts the feedback voltage into an electrostatic force acting on the proof mass and counterbalancing the inertial force. Figure 1 shows a generic EMΣΔM as a block diagram. Stability and performance are mainly dependent on the chosen architecture and the choice of the various gains in the pick-off circuitry and signal paths.

For our design methodology the user must first choose an architecture and the order, which can either be taken from the literature on EMΣΔM, an architecture for a purely electronic A/D ΣΔM, or a novel architecture developed by the user. The next step is to develop a Simulink model. The model can be as simple or as complex as deemed necessary by the user. Second order effects may be included. A few examples include: (i) the pick-off circuit can be modelled simply as a gain constant, or the nonlinear relationship between displacement and differential change in capacitance may be included; (ii) The micromachined sensing element may be simply modelled as a second order lumped parameter system with mass, damping and mechanical spring constant as the only parameters, or higher order modes e.g., from the dynamics of the sense fingers can be included [22]; (iii) The modulation of the electrostatic force by the residual motion of the proof mass [8] can be included. In principle, there is no limitation on the complexity of the Simulink model, however there is obviously a trade-off between simulation time and model complexity.

**Figure 2.**
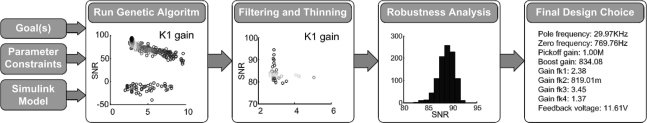
Generic process flow for the GA-based design algorithm.

The proposed methodology is represented by the flow-chart in Figure 2. Besides a parameterized Simulink model, the user is required to specify one or several goals for the GA to optimize as an objective. For closed loop MEMS sensors with a digital output typical objectives are: (i) the SNR, which should be maximized and is calculated based on the power spectral density of the output bitstream; (ii) the residual motion of the proof mass, which should be significantly smaller when compared to an open loop sensor. An unstable system can be determined from a negative or very low SNR so optimising towards high SNR solutions ensures that stability is addressed as part of the process. The ratio between open loop and closed loop proof mass deflection provides a measure of how well the sensing element is controlled by the electrostatic feedback force, and gives insight into the improvement in dynamic range compared to the open loop case. Furthermore, the GA requires a list of parameters it can change within user specified boundaries.

The GA is then initialized with a user specified number, N_R_ of, within the constraints, randomly chosen parameter sets; which is termed a population. Each parameter set is termed an individual. This initial population represents generation 1. The system then runs N_R_ simulations (one for each individual) and records the performance objectives for each individual, for example SNR and proof mass displacement as previously discussed. Once the first generation has been simulated, the result is stored as a table where each row consists of the parameter set for one individual and its performance. As explained in the previous section, the GA sorts the results and then performs a number of functions including picking the very best individuals (elite preservation), generating a certain number of new random individuals (mutation) and cross fertilising good individuals to create new offspring. This last step actually involves taking different parameters from different good individuals and combining them to create a new individual (child). These three steps create generation 2, which again consists of N_R_ individuals. The whole process continues until either a specified maximum number of generations has been reached or the user monitoring the evolution determines that sufficient convergence has been achieved. Although it would be possible to automate the convergence detection, for example by calculating bit string affinity, we have found in practice that the insight gained from making this an interactive decision is very valuable.

Simulation length is an important consideration during the GA process and introduces a trade-off between accuracy and total optimisation time. Often systems can appear initially stable, only to lose stability a short time later and therefore it is possible to unwittingly promote unstable systems forward in the evolutionary process if the simulation time is too short. However, long simulation times can result in excessively time-consuming optimisation periods, given the large number of simulations involved. This issue has been addressed in this work by typically running a small number of simulations initially to establish a ‘quick’ simulation period that represents a reasonable trade-off between the chance of missed instability and computation time. When the final solution is chosen at the end of the whole process, a more extensive simulation is performed with a ‘long’ simulation period to verify stability beyond doubt. Values for the ‘quick’ simulation periods will depend strongly on the type of architecture being designed but typically lie in the region of a few seconds, and the ‘long’ simulation period is typically 8 times longer than the ‘quick’ period. Both of these parameters are defined alongside all the goals, parameters and constraints in the system file.

The next step in the methodology is to consider robustness, which is an important measure of how parameter variation will affect performance or stability and a key contribution of this work. It cannot be assumed that the individuals in the final population of the GA step are the most robust, since they have only been optimized for SNR and RMS displacement, not tolerance to variation. For example, an individual in one of the earlier populations may have only slightly lower performance than one in the final population but may be far more robust. For this reason, the robustness stage of the process must consider the full history of individuals, which we refer to as a census. Theoretically, Monte Carlo simulations could be performed on every individual in the census, however, with hundreds of simulations per individual required for the robustness analysis this would be too time consuming.

Therefore, before Monte Carlo robustness analysis is performed, the census needs to be filtered to discard all individuals that do not meet the objectives (*i.e.*, the goals). After this filtering process, there may still exist a very large number of acceptable individuals, many of which may be close to each other in the design space. To run Monte Carlo simulations on two individuals which are themselves very similar would be inefficient. For this reason a thinning algorithm has been implemented which thins out the filtered set to give a smaller, more unique, and dispersed set of fit individuals. The thinning algorithm works by considering each individual in turn and adding an adjustable margin either side of the parameters for that solution. Any other individuals in the census whose parameters all lie within the margin of the individual under consideration are removed. After the census is traversed a smaller number of distinct design points are left. The algorithm repeats this whole process, whilst adjusting the separation margin, until the desired number of solutions remains. Monte Carlo simulations are then performed on each remaining individual using user specified standard deviation values for all electrical and mechanical parameters of the system model. The results from these statistical simulations are analysed to determine how many of the Monte Carlo simulations passed the goals for that individual, and from this a yield is calculated. After the robustness process is complete, the result is therefore a list of all feasible and optimal individuals and their simulated yields, and from this list the user can choose the final design solution.

## 3. Example 1: A 5th Order Low-Pass EMΣΔM for a MEMs Accelerometer

### 3.1. System Setup and Initialization

**Figure 3.**
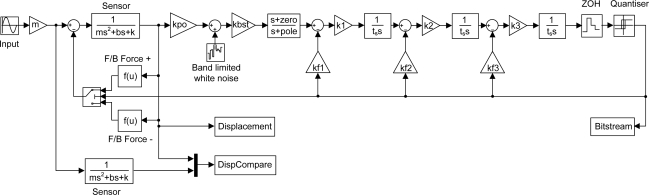
Simulink model of a 5th order EMΣΔM for a MEMS accelerometer.

To demonstrate the design procedure we present a 5th order low-pass ΣΔM for a MEMS accelerometer with a sensing element fabricated in SOI (Silicon on Insulator). The main specifications of the sensor are listed in Table 1 and represent typical values for a high performance MEMS accelerometer. The Simulink model, shown in Figure 3, is a 5th order EMΣΔM with distributed feedback architecture described in [13] albeit for a sensing element with different parameters. The model consists of a second order lumped parameter representation of the sensing element (which is duplicated to compare the open loop and closed loop proof mass deflection), an ideal capacitive position measurement circuit with pick-off gain kpo and associated white noise of the first amplifier (1/f noise is neglected here, but could easily be included), a boost gain kbst, a lead-lag compensator with a zero and a pole frequency as design parameters, three integrators with associated gains k1, k2 and k3, three feedback gains kf1, kf2 and kf3, a zero-order-hold, a 1 bit quantizer and the feedback arrangement in which an electrostatic force is acting on the proof mass in either positive or negative direction, depending on the quantizer state. During definition of the system architecture the designer must choose the level of model abstraction to implement for the individual blocks. For example, in the simplest case, gain blocks can be modelled as a multiplying constant, whereas a more complete approach may model bandwidth limitations in the form of a pole, and dynamic range limitations in the form of limiting functions. This represents another trade-off in the process, since an increase in model complexity clearly results in increased simulation times. In practice the authors have found that a high level of abstraction is sufficient for many system architectures.

In this example the ten parameters shown in Table 2 are assumed as design parameters which the GA will work on. The table also shows the range over which the parameters are varied. The lower and upper boundary need to be specified by the user and should be chosen by circuit implementation considerations; for example, typical values for the gain from proof mass displacement to voltage can be taken from the literature [8]. In fact, here we choose to vary the boost gain kbst, whereas the pick-off gain, kpo, representing the gain of the first amplifier, is assumed fixed at 400 kV/m.

For the ΣΔM the oversampling ratio (OSR) needs to be specified. The OSR is related to the sensor bandwidth, BW and the sampling frequency fs by OSR = fs/(2 × BW). Here, we choose OSR = 64 resulting in a sampling frequency of 128 kHz. Furthermore, the criteria (*i.e.*, the goal values) for the GA to optimize need to be defined; in this example these are the SNR (to be maximized) and the root mean square (RMS) value of the proof mass deflection (to be minimized). Here, we choose pass goal values of a SNR > 100 dB and the RMS proof mass deflection <40 nm. Finally, the GA needs to have values for the number of individuals in each generation, and the number of generations; we choose here 200 and 15, respectively. The choice of population size and number of generations is a trade-off between simulation time, and the degree of design space exploration and individual diversity that will be achieved during the GA evolution process. In practice we have found that a population size in the order of 20 times the number of design parameters represents a good trade-off. Progress of the evolution can be monitored in real time since the program streams text results to the Matlab command window. When the SNR performance changes very little from generation to generation, this is a good indicator that peak performance has been achieved. Therefore, a large number of generations are often specified and the GA process halted when it is visually clear that peak fitness has been reached. Following this method can significantly reduce overall simulation time.

### 3.2. Genetic Algorithm

The GA is then run using 200 individuals, which are, within the specified range, randomly chosen parameter sets; for each individual a simulation is carried out and the SNR is calculated. This calculation is performed by a function ‘calcSNR’ available through the Delta Sigma Toolbox for Matlab [16]. For each simulation a row of values is recorded and displayed in the Matlab command window representing the design parameters and goal function values. Table 3 shows three blocks of 10 individuals each; the first block for generation 1, the second for generation 8, and the third for generation 15, which is the last one in this example.

Examining the SNR column (2nd from the right), it can be seen that some SNR values are negative in the first block; this indicates an unstable system, as the SNR is a good indicator for system stability [13]. The last column is the proof mass deflection which in all cases of block 1 is higher than the specified goal value (<40 nm); in fact the values are physically impossible as they are larger than the electrode gap. Such large deflections are possible due to the output signal of the sensor block in the Simulink model not being limited to the physical constraints. Although model refinement could easily be added, in practice it is not necessary since the goal of minimising RMS displacement halts the evolution of these solutions. Not surprisingly, none of the randomly chosen individuals of generation 1 yields a working system. When examining the second block of 10 individuals belonging to generation 8, it can be seen that about half are still unstable, but there are now also some solutions which meet the specified SNR value and maximum allowed proof mass deflection. In the final block (generation 15) all but one individual meet the specified goal values, but there is still one which represents an unstable system as it has a negative SNR.

### 3.3. Robustness Analysis

**Figure 4.**
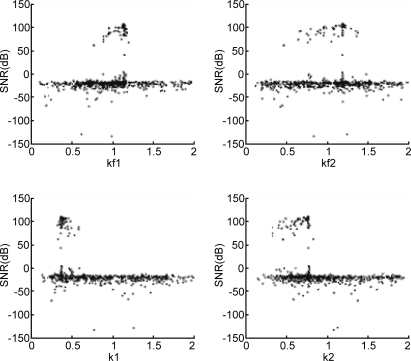
Scatter plot for the EMΣΔM gain constants (k1–k2 and kf1–kf2) from the entire GA set of individuals including unfeasible designs.

**Figure 5.**
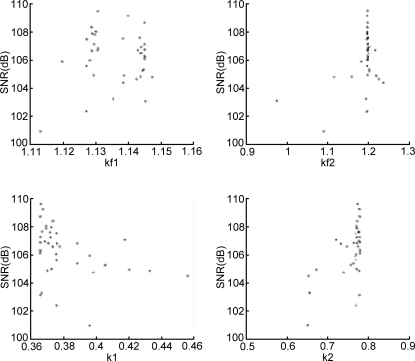
Scatter plot for the EMΣΔM gain constants (k1–k2 and kf1–kf2) of the 40 individuals remaining after thinning and filtering.

The next step in the design process is robustness analysis which starts with a thinning and filtering algorithm. All individuals of all generations are stored in a matrix with 200 × 15 rows (number of individuals times number of generations). For the robustness analysis the same goal function values (a minimum SNR of 100 dB and a maximum RMS deflection of 40 nm) are chosen; if required these values can be modified at this point. The filtering algorithm simply discards the individuals that have a SNR < 100 dB or an RMS proof mass deflection >40 nm. The thinning algorithm now finds the most distinct individuals in the remaining design space, as explained in Section 2. Figures 4 and 5 show scatter plots of the design parameters (for brevity only four gain constants are shown) before and after the filtering and thinning algorithm, respectively.

**Figure 6.**
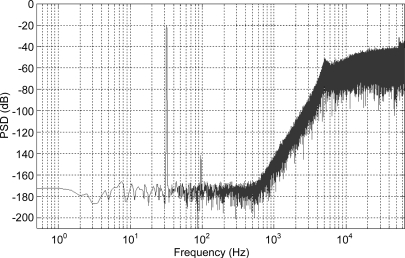
Power spectral density of the individual chosen as final solution.

The number of individuals to be considered for the robustness analysis is specified as a user defined parameter, and is set to 40 here. Another user defined parameter sets the number of Monte Carlo simulations that will be performed for each individual; in this example 100. For each design parameter the user specifies a standard deviation providing a measure of parameter variation. The robustness analysis typically varies more design parameters than those explored by GA; for example the parameters of the sensing elements (its mass, damping coefficient and spring constant) were considered as fixed for the GA, but for the robustness analysis were varied by 2%, 25% and 5%, respectively, whereas the electronic gain constants optimized by the GA were varied only by 2%. This reflects the considerable fabrication tolerances that a micromachined sensing element typically exhibits. A function in the program generates 100 Gaussian distributed parameter sets based on a particular individual’s parameters (as the means) and the user supplied standard deviations. For each individual therefore, 100 simulations are run and the SNR and RMS displacement performance recorded. A yield value is calculated representing the percentage of the simulations for each individual that exceed the specified goal values. The user can then review the yield and performance of the investigated individuals and choose one as the final design. Here, the final parameter set is shown in Table 4; it has a SNR of 109.21 dB, an RMS proof mass displacement of 31 nm and a yield of 68%. Figure 6 shows the PSD of the output bitstream for these design parameters.

## 4. Example 2: A 6th Order Band-Pass EMΣΔM for a MEMs Gyroscope

A further example of the proposed design methodology is now presented for a continuous time, 6th order band-pass EMΣΔM for a vibratory rate MEMS gyroscope fabricated in SOI technology, as described in [23]. Continuous time, band-pass EMΣΔM are a relatively recent development and are particularly difficult to design as the electrical filter part consists of resonators requiring both return-to-zero (RZ) and half-delay return-to-zero (HZ) digital to analogue conversion [14,24]. The mechanical parameters of the gyroscope are listed in Table 5 along with the main ΣΔM specifications.

**Figure 7.**
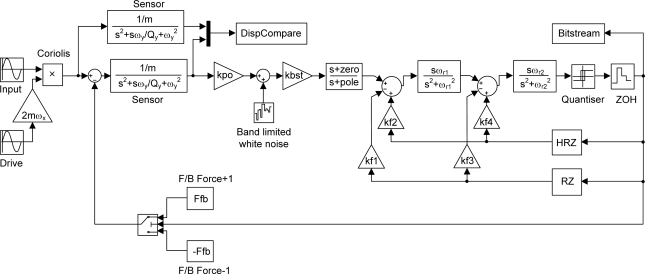
Simulink model of a six order continuous time, band-pass EMΣΔM for the sense mode of a MEMS gyroscope.

The Simulink model is shown in Figure 7 and consists of a second order lumped model of the sensor, which is again duplicated to compare the open loop and closed loop proof mass deflection. The model includes an ideal pickoff circuit with gain kpo and associated white noise, a boost gain kbst, a lead-lag compensator, a zero-order-hold and 1 bit quantizer, four local feedback gains kf1 to kf4, and an electrostatic force feedback arrangement which acts on the proof mass in a direction depending on the quantizer output state.

**Figure 8.**
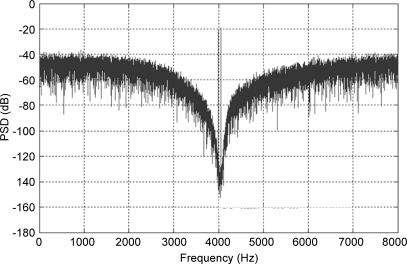
Power spectral density of the final gyroscope solution.

The genetic algorithm is then executed, which again creates and simulates an initial population of individuals, determining their fitness by combining their SNR and sense mode proof mass displacement. A combination of cross fertilisation, mutation, and elite preservation is used to create a new population and the evolution continues. After the specified number of generations, the algorithm halts, storing the entire multi generation census for the next step. Filtering and thinning is performed on the census results to ignore individuals which do not pass the specified goal values of 70 dB SNR and 20 nm RMS displacement, or are too close to one another. The individuals remaining after the thinning algorithm are feasible and distinct solutions, and are then used as the input to the Monte Carlo based robustness analysis of the next stage. A total of 200 Monte Carlo simulations are performed on each of these solutions using realistic standard deviations for all electrical and mechanical parameters, and from this a simulated yield is calculated and documented against the solution point. The designer then has the opportunity to choose a solution from this final list, trading off performance against yield for their particular application. In this case the parameters for the chosen design are shown in Table 6. A lengthy transient simulation is then performed to obtain the PSD of the output signal, which is shown in Figure 8. The solution performs well with a SNR of 92.4 dB within the 64 Hz signal bandwidth and as we expect, there is a pronounced band-pass noise shaping around the signal band.

## 5. Discussion

The two examples presented illustrate the usefulness of the methodology for the design of arbitrarily complex EMΣΔM. To the best of the authors’ knowledge this is the first time such a design methodology has been presented based on non-linear models. The design methodology for the majority of EMΣΔM presented in the literature is not described, which is an indication that a manual process relying on trial and error was used. This requires a considerable experience in ΣΔM and MEMS sensor design and hence has a high initial knowledge threshold. Even after sufficient knowledge has been gained it can often take weeks to develop a satisfactory system design, with no real certainty that the system is robust or even optimal. The proposed approach greatly expedites the design process and gives much greater confidence that the results are both optimal and robust. In some literature sources a design methodology for EMΣΔM is described based on root locus techniques [13] or directly on the transfer function [25]. However, this approach has two disadvantages: (i) it relies on a linearized model of the quantizer consisting of white quantisation noise and a quantizer gain which has limited validity since it does not consider the non-linear term introduced due to the dependence of the feedback force on the sensor mass position; and (ii) the linearized model is typically used to predict only the performance of the EMΣΔM, rather than its stability. The systematic approach proposed in [11] is also based on linear system analysis so suffers the same drawbacks and also does not consider tolerance to parameter variation which can easily lead to an unstable system. The designer is therefore left with an uncertainty as to how close the chosen parameter set is to the optimum solution that is robust in practice.

The design methodology described here circumvents both drawbacks: it is based on a full non-linear system model and it yields a design solution that is very close to the optimum, as it takes into account both SNR and proof mass displacement as performance measures. Additional performance parameters, such as dynamic range and maximum input signal could be additionally included as optimization metrics as required. Another advantage is the designer’s total freedom in the initial choice of the control system architecture; whereas the EMΣΔM described in the literature to date all are adapted architectures of ΣΔM analogue to digital converters. Therefore, our methodology facilitates the exploration of novel architectures for EMΣΔM; one example is to have a two-channel ΣΔM for the sense mode of a gyroscope, one channel for the signal, the other for the quadrature error. Furthermore, the GA design parameter set could be extended such that different architectures, or loop orders could be available as part of the GA evolution, allowing extremely diverse design space exploration.

The robustness analysis performed following the GA is a key contribution of the work, giving confidence in a design and ensuring manufacturability. Without this it is possible to design a system which may easily become unstable due to inevitable fabrication tolerances. As with any multi objective optimisation, there is no single optimal solution but instead a range of equally optimal solutions, which is why it is important for the designer to choose the final design solution based on a performance *versus* yield trade-off in the final step. This final solution can then be implemented in hardware, using standard circuit techniques, and hence is not discussed here; the reader is referred to e.g., [6,7,9,10].

Many design flows have been performed by the authors for a wide range of EMΣΔM architectures and they are confident to claim that the GA explores the design space well and finds an excellent design solution even for complex and non-linear design spaces with multiple objectives. A side benefit of the approach is the insight gained from its use by those who have little experience in the area. With typical design times of a single day for complex architectures, the approach offers an extremely efficient alternative to manual design procedures which often take weeks.

## 6. Conclusions

The presented methodology allows the system level design of arbitrarily complex EMΣΔM with ease and in a short period of time. The design process relies on a GA that varies a set of system parameters and records the performance for each set. After a filtering and thinning step a robustness analysis is carried out to ensure system stability in the presence of fabrication tolerances, which can be considerable especially for micromachined sensors. The usefulness of the approach has been illustrated through two design examples including a 5th order low-pass EMΣΔM for a MEMS accelerometer, and a 6th order band-pass EMΣΔM for the sense mode of a MEMS gyroscope. In both cases the described methodology delivers near optimum system level design parameters. Compared with previously described design of EMΣΔM our methodology provides the users with greater confidence that the final design solution is near optimum and robust, ensuring stability in the presence of fabrication tolerances.

## Figures and Tables

**Table 1.** MEMS Accelerometer Parameters.

**Parameter**	**Value**
Mass [kg]	1.7e−6
Damping coefficient [N/ms]	3.5e−4
Spring constant [N/m]	5.5
Nominal capacitance [pF]	5.5
Nominal electrode gap [um]	6
Bandwidth [kHz]	1
Max. acceleration [G]	+/−2.5 G

**Table 2.** Design Parameters for the Genetic Algorithm.

**GA design parameter**	**Parameter range**
Boost gain kbst [V/V]	20–400
Minor feedback loop gain kf1 [V/V]	0.1–2
Minor feedback loop gain kf2 [V/V]	0.1–2
Minor feedback loop gain kf3 [V/V]	0.1–2
Integrator gain k1 [V/V]	0.1–2
Integrator gain k2 [V/V]	0.1–2
Integrator gain k3 [V/V]	0.1–2
Feedback voltage [V]	10–30
Compensator zero frequency [kHz]	0.5–50
Compensator pole frequency [kHz]	10–1,000

**Table 3.** Example Individuals in the Evolutionary Process.

**Individual/generation**		**Genetic algorithm design parameters**										**Goal functions**	

		**kbst (V/V)**	**kf1 (V/V)**	**kf2 (V/V)**	**kf3 (V/V)**	**k1 (V/V)**	**k2 (V/V)**	**k3 (V/V)**	**Zero (Hz)**	**Pole (Hz)**	**kf1 (V/V)**	**SNR (dB)**	**Disp. (μm)**
**Block 1: Individuals 50–59 of Generation 1**	50/1	234.49	0.96328	0.97271	1.13	1.97	1.21	0.70887	14.42	9,160	200,632	−19.21	326.20
	51/1	94.01	0.85257	1.91	1.33	1.1	0.68428	1.17	25.96	8,819	163,056	−19.11	920.04
	52/1	155.32	1.37	1.8	0.43793	1.22	1.23	1.47	22.28	27,150	139,900	−22.79	846.42
	53/1	96.58	1.84	1.52	0.71009	0.57052	1.19	0.52997	25.5	46,26	391,552	−25.01	852.42
	54/1	109.55	1.76	1.88	0.33214	0.72423	1.86	1.21	16.43	10,567	497,281	−22.71	1,030.00
	55/1	195.72	0.4096	0.69417	0.17444	0.90656	1.49	1.63	13.92	24,260	230,138	−20.5	856.28
	56/1	109.55	1.76	1.88	0.33214	0.72423	1.86	1.21	16.43	10,567	497,281	−22.71	1,030.00
	57/1	51.13	0.54297	1.93	0.86304	1.28	1.08	1.64	15.49	1,664	98,432	−30.21	234.18
	58/1	114.42	1.94	1.15	1.79	0.74525	1.77	1.72	25.95	11,089	503,748	−22.29	451.83
	59/1	173.05	1.61	0.42045	1.75	1.37	1.81	0.99297	12.17	9,593	860,350	−26.48	634.67

**Block 2: Individuals 50–59 of Generation 8**	50/8	189.17	0.87123	1.61	0.28068	0.7491	0.65026	1.31	20.68	2,301	130,096	−29.06	321.07
	51/8	183.63	1.07	1.36	1.32	0.96871	0.70766	0.96289	23.03	4,139	51,573	−20.11	66.59
	52/8	205.03	1.15	1.2	1.75	0.36596	0.78318	0.87712	23.05	5,466	96,872	104.63	0.0342
	53/8	205.03	1.15	1.2	1.75	0.36596	0.78318	0.87712	23.05	5,466	96,872	104.63	0.0342
	54/8	205.03	1.15	1.2	1.75	0.36596	0.78318	0.87712	23.05	5,466	96,872	104.63	0.0342
	55/8	185.93	0.56781	0.72001	1.92	0.64756	0.8026	1.16	13.05	13,159	412,664	−16	82.72
	56/8	22.41	0.59207	0.20479	1.59	0.25323	1.62	1.47	10.09	5,441	242,054	−23.79	11.16
	57/8	27.41	0.7697	0.47903	1.58	0.32926	0.4721	1.44	13.98	5,234	251,351	60.91	0.8161
	58/8	92.89	0.8883	1.49	1.7	0.58791	0.61525	0.67127	18.02	4,174	309,443	−1.46	193.27
	59/8	148.94	0.9982	1.53	0.55979	1.66	0.57027	1.41	20.18	4,478	50,853	−16.32	63.21

**Block 3: Individuals 50–59 of Generation 15**	50/15	205.03	1.15	1.2	1.75	0.36596	0.78318	0.87712	23.05	4,442	78,727	109.24	0.03086
	51/15	205.03	1.15	1.2	1.75	0.36596	0.78318	0.87712	23.05	4,423	78,390	109.21	0.03096
	52/15	205.03	1.15	1.2	1.75	0.36596	0.9726	0.87712	23.05	4,508	79,906	−16.5	57.76
	53/15	203.02	1.13	1.2	1.75	0.37593	0.77241	0.87096	23.05	4,404	78,053	109.15	0.03076
	54/15	203.02	1.13	1.2	1.75	0.37593	0.77241	0.87096	23.05	4,551	80,664	108.15	0.03076
	55/15	203.02	1.13	1.2	1.75	0.37593	0.77241	0.87096	23.05	4,551	80,664	108.15	0.03076
	56/15	203.78	1.14	1.2	1.75	0.3703	0.78191	0.87604	23.05	4,162	73,759	107.81	0.03092
	57/15	203.78	1.14	1.2	1.75	0.3703	0.78191	0.87604	23.05	4,162	73,759	107.81	0.03092
	58/15	205.03	1.15	1.2	1.75	0.36596	0.78318	0.87477	23.05	5,252	93,083	107.18	0.03228
	59/15	205.03	1.15	1.2	1.75	0.36596	0.78318	0.87712	23.05	4,420	78,348	109.19	0.03096

**Table 4.** Final Design Parameters.

**GA design parameter**	**Parameter value**
Boost gain kbst [V/V]	204.92
Minor feedback loop gain kf1 [V/V]	1.14
Minor feedback loop gain kf2 [V/V]	1.2
Minor feedback loop gain kf3 [V/V]	1.75
Integrator gain k1 [V/V]	0.37
Integrator gain k2 [V/V]	0.78
Integrator gain k3 [V/V]	0.87
Feedback voltage [V]	23.05
Compensator zero frequency [kHz]	4.413
Compensator pole frequency [kHz]	78.22

**Table 5.** Gyroscope and ΣΔM Parameters.

**Parameter**	**Drive mode**	**Sense mode**
Mass of proof mass [kg]	2e−6	2e−6
Mechanical spring constant [N/m]	1,268	1,328
Resonant frequency [Hz]	4,027	4,073
Quality factor	216	85
Pick-off gain [V/m]	-	1e6
Sampling frequency [Hz]	-	32,768
Oversampling ratio	-	256
Frequency of input angular rate [Hz]	-	32
Max. input angular rate [°/s]	-	200

**Table 6.** Design Parameters for the Genetic Algorithm.

**GA design parameter**	**Parameter range**
Boost gain, kbst [V/V]	834.08
Minor feedback loop gain kf1 [V/V]	2.38
Minor feedback loop gain kf2 [V/V]	0.819
Minor feedback loop gain kf3 [V/V]	3.45
Minor feedback loop gain kf4 [V/V]	1.37
Feedback voltage [V]	11.61
Compensator zero frequency [Hz]	769
Compensator pole frequency [Hz]	29,970

## References

[1] Lemkin M, Boser BE (1999). A three-axis micromachined accelerometer with a CMOS position-sense interface and digital offset-trim electronics. IEEE J. Solid-State Circuits.

[2] Jiang X, Seeger JI, Kraft M, Boser BE A monolithic surface micromachined Z-axis gyroscope with digital output.

[3] Kraft M, Lewis CP, Hesketh TG (1998). Closed loop silicon accelerometers. IEEE Proc. Circuits Devices Syst.

[4] Norsworth SN, Schreier R, Temes C (1997). Delta-Sigma Data Converters, Theory, Design, and Simulation.

[5] Smith T, Nys O, Chevroulet M, DeCoulon Y, Degrauwe M A 15 b electromechanical Sigma-Delta converter for acceleration measurements.

[6] Kajita T, Moon U-K, Temes GC (2002). A two-chip interface for a MEMS accelerometer. IEEE Trans. Instrum. Meas.

[7] Dong Y, Kraft M, Gollasch CO (2005). A high performance accelerometer with fifth order sigma delta modulator. J. Micromech. Microeng.

[8] Dong Y, Kraft M, Redman-White W (2006). Force feedback linearization for higher-order electromechanical Sigma-Delta modulators. J. Micromech. Microeng.

[9] Petkov V, Boser BE (2005). A fourth-order interface for micromachined inertial sensors. IEEE J. Solid State Circuits.

[10] Dong Y, Kraft M, Hedenstierna N, Redman-White W (2008). Microgyroscope control system using a high-order band-pass continuous-time sigma-delta modulator. Sens. Actuat. A.

[11] Raman J, Rombouts P, Weyten L (2008). An unconstrained architecture for systematic design of higher order Sigma Delta force-feedback loops. IEEE Trans. Circuits Syst. I.

[12] Raman J, Cretu E, Rombouts P, Weyten L (2009). A closed-loop digitally controlled MEMS gyroscope with unconstrained sigma-delta force-feedback. IEEE Sens. J.

[13] Dong Y, Kraft M, Redman-White W (2007). High order noise shaping filters for high performance inertial sensors. IEEE Trans. Instrum. Meas.

[14] Dong Y, Kraft M, Redman-White W (2007). Micromachined vibratory gyroscopes controlled by a high order band-pass sigma delta modulator. IEEE Sens. J.

[15] Ardalan S, Paulos J (1987). An analysis of nonlinear behavior in Delta-Sigma modulators. IEEE Trans. Circuits Syst.

[16] Schreier R Delta Sigma Toolbox, MatLab File Exchange.

[17] Lota J, Al-Janabi M, Kale I (2008). Nonlinear-stability analysis of higher order Δ-Σ modulators for DC and sinusoidal inputs. IEEE Trans. Instrum. Meas.

[18] Deb K (2001). Multi-Objective Optimization Using Evolutionary Algorithms.

[19] Horn J, Nafpliotis N, Goldberg D A niched pareto genetic algorithm for multiobjective optimization.

[20] Deb K, Pratap A, Agarwal S, Meyarivan T (2002). A fast and elitist multiobjective Genetic Algorithm: NSGA-II. IEEE Trans. Evol. Comput.

[21] Engesser M, Buhmann A, Franke AR, Korvink JG (2009). Efficient reliability-based design optimization for microelectromechanical systems. IEEE Sens. J.

[22] Seeger JI, Jiang X, Kraft M, Boser BE Sense finger dynamics in a ΣΔ force feedback gyroscope.

[23] Ding H, Liu X, Cui J, Chi X, Lin L, Kraft M, Yang Z, Yan G (2010). A high-resolution Silicon-on-glass Z-axis gyroscope operating at atmospheric pressure. IEEE Sens. J.

[24] Maurino R, Mole P (2000). A 200 MHz IF 11-bit fourth-order bandpass sigmadelta ADC in SiGe. IEEE J. Solid State Circuits.

[25] Luo J, Ding H, Kraft M A new design methodology for electro-mechanical Sigma-Delta modulators.

